# Intronic Variants in the *NFKB1* Gene May Influence Hearing Forecast in Patients with Unilateral Sensorineural Hearing Loss in Meniere's Disease

**DOI:** 10.1371/journal.pone.0112171

**Published:** 2014-11-14

**Authors:** Sonia Cabrera, Elena Sanchez, Teresa Requena, Manuel Martinez-Bueno, Jesus Benitez, Nicolas Perez, Gabriel Trinidad, Andrés Soto-Varela, Sofía Santos-Perez, Eduardo Martin-Sanz, Jesus Fraile, Paz Perez, Marta E. Alarcon-Riquelme, Angel Batuecas, Juan M. Espinosa-Sanchez, Ismael Aran, Jose A. Lopez-Escamez

**Affiliations:** 1 Otology & Neurotology Group CTS495, Department of Genomic Medicine- Centro de Genómica e Investigación Oncológica – Pfizer/Universidad de Granada/Junta de Andalucía (GENYO), Granada, Spain; 2 Department of Neurology, Icahn School of Medicine at Mount Sinai, New York City, New York, United States of America; 3 Group of Genetics of Complex Diseases, Department of Genomic Medicine - Centro de Genómica e Investigación Oncológica – Pfizer/Universidad de Granada/Junta de Andalucía (GENYO), Granada, Spain; 4 Department of Otolaryngology, Hospital Universitario de Gran Canaria Dr Negrin, Las Palmas, Spain; 5 Department of Otolaryngology, Clinica Universidad de Navarra, Pamplona, Spain; 6 Division of Otoneurology, Department of Otorhinolaryngology, Complejo Hospitalario Badajoz, Badajoz, Spain; 7 Division of Otoneurology, Department of Otorhinolaryngology, Complexo Hospitalario Universitario, Santiago de Compostela, Spain; 8 Department of Otolaryngology, Hospital Universitario de Getafe, Getafe, Spain; 9 Department of Otolaryngology, Hospital Miguel Servet, Zaragoza, Spain; 10 Department of Otorhinolaryngology, Hospital Cabueñes, Gijón, Spain; 11 Department of Otolaryngology, Hospital Universitario Salamanca, Salamanca, Spain; 12 Department of Otorhinolaryngology, Hospital San Agustin, Linares, Jaen, Spain; 13 Department of Otolaryngology, Complexo Hospitalario de Pontevedra, Pontevedra, Spain; 14 Department of Otolaryngology, Hospital de Poniente, El Ejido, Almería, Spain; University of Sydney, Australia

## Abstract

Meniere's disease is an episodic vestibular syndrome associated with sensorineural hearing loss (SNHL) and tinnitus. Patients with MD have an elevated prevalence of several autoimmune diseases (rheumatoid arthritis, systemic lupus erythematosus, ankylosing spondylitis and psoriasis), which suggests a shared autoimmune background. Functional variants of several genes involved in the NF-κB pathway, such as *REL*, *TNFAIP3*, *NFKB1* and *TNIP1*, have been associated with two or more immune-mediated diseases and allelic variations in the *TLR10* gene may influence bilateral affectation and clinical course in MD. We have genotyped 716 cases of MD and 1628 controls by using the ImmunoChip, a high-density genotyping array containing 186 autoimmune loci, to explore the association of immune system related-loci with sporadic MD. Although no single nucleotide polymorphism (SNP) reached a genome-wide significant association (p<10^−8^), we selected allelic variants in the NF-kB pathway for further analyses to evaluate the impact of these SNPs in the clinical outcome of MD in our cohort. None of the selected SNPs increased susceptibility for MD in patients with uni or bilateral SNHL. However, two potential regulatory variants in the *NFKB1* gene (rs3774937 and rs4648011) were associated with a faster hearing loss progression in patients with unilateral SNHL. So, individuals with unilateral MD carrying the C allele in rs3774937 or G allele in rs4648011 had a shorter mean time to reach hearing stage 3 (>40 dB HL) (log-rank test, corrected p values were p = 0.009 for rs3774937 and p = 0.003 for rs4648011, respectively). No variants influenced hearing in bilateral MD. Our data support that the allelic variants rs3774937 and rs4648011 can modify hearing outcome in patients with MD and unilateral SNHL.

## Introduction

Meniere's disease (MD) is a chronic disorder affecting the inner ear characterized by fluctuating sensorineural hearing loss (SNHL), episodes of vertigo, tinnitus, and aural fullness and it can affect both ears in 10–40% of cases [1]. The etiology and pathogenesis remain unknown, although one-third of MD cases may have an aberrant response of the adaptive or innate immune system, the immunological mechanisms involved have not been investigated [2].

Several mechanisms may explain the development of immune-mediated inner ear disease (IED): a) cross-reactions with a cross-reactive epitope (antibodies cause accidental inner ear damage because the ear shares epitopes with a potentially harmful substance, virus or bacteria) as suspected for some inflammatory diseases [3], b) damage to the inner ear caused by pro-inflammatory cytokines such as IL-1B [4], [5] or TNF [6] as in some autoimmune diseases, or c) inappropriate immune response or intolerance to harmless unrecognized substances combined with genetic factors that modify the immune response as in allergies [2].

IED and MD may have an overlapping phenotype and autoimmune mechanisms could be associated with the pathophysiology of MD. Some evidence support this hypothesis including the response to steroids therapy, the finding of elevated levels of autoantibodies or circulating immune complexes (CIC) in the serum of some patients with MD against inner ear antigens and the association of allelic variants of MHC class I polypeptide-related sequence A (MICA) and Toll-like receptor 10 (*TLR10*, rs11096955) gene with hearing loss progression in patients with MD [7], [8].

These findings together with the elevated prevalence of several autoimmune diseases such as rheumatoid arthritis (RA), systemic lupus erythematosus (SLE), ankylosing spondylitis (AS), and psoriasis in patients with MD, suggest an autoimmune component [9], [10].

Nuclear factor κB (NF-κB) is a crucial pleiotropic transcription factor (TF) which regulates inflammation and the innate and adaptive immune response [11]. Five members of this transcription factor family have been identified: RELA (p65), RELB, REL (c-Rel), NF-κB1 (p105) and NF-κB2 (p100). Of note, p105 and p100 are pro-forms proteolytically processed to p50 and p52 [12]. All members of the NF-κB family harbor an N-terminal Rel homology domain (RHD), which interacts with DNA elements and mediates homo- and heterodimerization. The complex p65–p50 is the most abundant form of heterodimer of the NF-κB family and it keeps in an inactive state in the cytoplasm bound to proteins of the IkB family, which are inhibitors of NF-κB [13].

In its canonical pathway, excitatory signaling can be mediated through Toll-like receptors (TLRs), Interleukin-1 receptor (IL-1R), tumor necrosis factor receptor (TNFR) and antigen receptors. Several genes involved in the regulation of NF-κB pathway have been associated with autoimmune disorders. So, ubiquitin-conjugating enzyme E2L3 (UBE2L3), which ubiquitylates p105 for its degradation, tumor necrosis factor alpha-induced protein 3 (TNFAIP3, also known as A20), an ubiquitin-editing enzyme with determines NF-κB activity or TNFAIP3-interacting protein (TNIP1) which inhibits TNF-induced NF-κB -dependent gene expression have genetic variants associated with several autoimmune diseases [14].

The genotyping of large cohorts of patients with several autoimmune diseases in genome-wide association studies has shown that most of these diseases share multiple susceptibility loci (www.immunobase.org) [14]. Among them, several genes in the NF-κB pathway are associated with two or more immune-mediated diseases, such as inflammatory bowel disease (*REL*, *TNFAIP3* and *NFKB1*), psoriasis (*REL*, *TNFAIP3*, *NFKB1* and *TNIP1*), coeliac disease (*REL* and *TNFAIP3*), rheumatoid arthritis (*REL* and *TNFAIP3*), type 1 diabetes (T1D) (*TNFAIP3*), systemic lupus erythematous (SLE) (*TNFAIP3* and *TNIP1*), multiple sclerosis and primary biliary cirrhosis (*NFKB1*) (Table 1).

**Table 1:** Single nucleotide polymorphisms in the NF-κB pathway with reported associations.

Chr	Position^a^	rsID	GENE (variant type)	Phenotype Association	P-Value	Reference
**6**	138199417	rs610604	*TNFAIP3* (intron)	Psoriasis	5.53×10^−5^	[39]
**5**	150440097	rs2233287	*TNIP1*(intron)	Asthma, systemic sclerosis	0.039, 6.17×10^−4^	[40], [41]
**5**	150438988	rs1422673	*TNIP1*(intron)	Asthma	0.011	[40]
**2**	61136129	rs13031237	*REL* (intron)	Rheumatoid Arthritis	7.29×10^−3^	[42]
**22**	21939675	rs5754217	*UBE2L3* (intron)	SLE	0.012	[43]
**22**	21917190	rs131654	*UBE2L3* (intron)	SLE	1.12×10^−7^	[44]
**22**	21932068	rs181362	*UBE2L3* (intron)	HDL cholesterol	3.72×10^−4^	[45]
**4**	103434253	rs3774937	*NFkB1* (intron)	Primary biliary cirrhosis, Body Weight	1.5×10^−10^ 0.041	[46, dbGaP^b^]
**4**	103475444	rs4648011	*NFkB1* (intron)	Body Weight	0.040	[dbGaP^b^]

a NCBI human genome build 37 coordinates.

b http://www.ncbi.nlm.nih.gov/gap [38].

We have used the ImmunoChip, a high-density genotyping array which includes 186 loci previously associated with 12 autoimmune diseases, to explore the association of these loci with MD and to evaluate the role of functional variants of genes involved in the NF-κB pathway with MD and their potential effect on the hearing outcome of the disease [14].

## Materials and Methods

### Study samples

This study was approved by the ethics committees of all the recruiting centers and all participating individuals gave written informed consent. This study was approved by the ethics committee for clinical research from Almería (Comité Ético de Investigación provincial de Almería), Jaén (Comité Ético de Investigación provincial de Jaén), Galicia (Comité Autonómico de Ética de la Investigación de Galicia), Asturias (Comité Ético de Investigación Clínica Autonómico de Asturias), Las Palmas (CEIC del Hospital de Gran Canaria Dr. Negrin), Navarra (Comité Ético de Investigación Clínica Autonómico de Navarra), Extremadura (Comité Ético Autonómico de Extremadura), Madrid-Getafe (CEIC Área 10 - Hospital Universitario de Getafe), Aragón (Comité Ético de Investigación Clínica de Aragón) and Salamanca (Comité Ético de Investigación Clínica Área de Salud de Salamanca). All the procedures described were performed in accordance with the highest ethical standards on human experimentation, the Helsinki Declaration of 1975.

We recruited a total of 716 patients who were diagnosed with definite MD according to the diagnostic scale for MD of the American Academy of Otolaryngology Head and Neck Surgery (AAO-HNS) [15] and 1628 control volunteers in a case–control study.

The diagnosis of MD was established according to the clinical guidelines defined by the Committee on Hearing and Equilibrium of the AAO-NHS in 1995 [15]. A complete neuro-otological evaluation including otoscopy, a pure-tone audiometry, nystagmus examination and caloric testing was carried out in all cases. Moreover, the protocol of diagnosis included a brain MRI to exclude other possible causes of neurological symptoms. Patients were followed with serial audiograms at each visit to monitor hearing loss from the initial diagnosis. The following clinical variables were studied in our series: gender, age, hearing stage, duration of the disease, bilateral SNHL, age of onset, type of headache, history of autoimmune disease, smoking, Tumarkin crisis and the functional scale of the AAO-HNS. Hearing staging was calculated by the audiogram obtained the day of inclusion for each patient with definite MD and was defined as the mean of four-tone average of 0.5, 1, 2 and 3 kHz according to the AAO-HNS criteria: stage 1, ≤25 dB; stage 2, 26–40 dB; stage 3, 41–70 dB; stage 4, >70 dB.

### DNA extraction and genotyping

Blood samples from each subject were collected and genomic DNA was isolated from peripheral blood leukocytes using the QIAamp DNA Mini Kit (Quiagen), according to the manufacturer's instructions. All genomic DNA was re-suspended in nuclease free water for the following study.

The concentration of genomic DNA was measured using the Qubit dsDNA BR Assay Kit (Invitrogen) and concentrations were standardized to 50 ng/µL for genotyping. All samples were genotyped using the ImmunoChip, a custom Illumina Infinium high density genotyping array containing 196524 markers across 186 known autoimmunity risk loci [14],in the iScan genotyping platform (Illumina Inc., San Diego, CA).

### Quality controls

Samples were clustered together by using the Illumina Genome Studio algorithm. Clusters were manually inspected and verified, removal of single nucleotide polymorphisms (SNPs) with poor clustering quality metrics (call frequency <0.98, cluster separation <0.4) was performed, and all SNPs with GenCall scores less than 0.15 were excluded. Quality controls (QC) were applied for all individuals and genotyped SNPs by using PLINK software (version 1.07) [16].

Samples with a genotype success rate of <90% were excluded from the analysis. The remaining samples were then evaluated for duplicates or related individuals and one individual from each pair was removed if the proportion of alleles share identical by descent (IBD) >0.5. Samples with increased heterozygosity rate (<0.18 and > 0.45) and missing data between cases and controls *P* value <10^−5^, were then removed from the analysis. Finally, genetic outliers determined by principal component analysis (PCA) were removed from the analysis (> 3 standard deviation around the mean). All familial cases were also excluded.

All the SNPs that did not meet the following criteria were excluded from further analysis: genotype success rate <90%, minor allele frequency (MAF) <5%, Hardy-Weinberg equilibrium <10^−4^ in controls and missing-genotype rate <0.5%. All markers in chromosome X were also excluded. After QC, 96899 SNPs remained with a MAF>0.05 for statistical analysis.

### Statistical analysis

After all QC, 689 cases (521 unilateral, 168 bilateral) and 1475 controls remained for further analyses. We have evaluated the association between each SNPs and patients with uni or bilateral MD. Allelic and genotype frequencies were compared between patients and controls by using χ2 test and calculating the odds ratios (OR) and 95% confidence intervals (CIs) using PLINK (version 1.07). P-values were adjusted by genomic control. The power was computed as the probability of detecting an association at the 0.05 significance level, assuming an OR = 1.5 (small effect size). Power analysis was estimated using the Quanto v1.2.4 software (Department of Preventive Medicine University of Southern California, CA, USA).

Moreover, among the SNPs compared above, we selected those SNPs which have been previously associated with other immune-mediated diseases, in the following genes: *NFKB1* (rs3774937, rs4648011), *REL* (rs13031237), *UBE2L3* (rs5754217, rs131654, rs181362), *TNFAIP3* (rs610604) and *TNIP1* (rs2233287, rs1422673) (Table 1). To assess if any of these variants have any effect on the clinical course of patients with MD. The median time to reach hearing loss >40 dB (hearing stage 3 or 4) for each genotype/allele was calculated according to the Kaplan–Meier method and survival curves were compared using the log-rank test (IBM SPSS Statistics 20.0). The *p* values were corrected, according to the Bonferroni's method, for the number of comparison made for each gene. p<0.05 was considered statistically significant.

### Validation of rs3774937 and rs4648011 genotyping

Case samples were also genotyped for two SNPs in the *NFKB1* gene (rs3774937 and rs4648011) with a TaqMan 5′ allelic discrimination assay according to manufactures' instructions (Life Technologies). Amplifications were performed in an ABI 7500 Fast Real-Time PCR System (LT) for continuous fluorescence monitoring. The alleles were determined using the SDS 2.2.1 software (LT). Functional evaluation of these regulatory variants was performed *in silico* by using the bioinformatics tools HaploReg (http://www.broadinstitute.org/mammals/haploreg/haploreg.php), seeQTL (http://www.bios.unc.edu/research/genomic_software/seeQTL/) and RegulomeDB (http://regulomedb.org/) to explore annotations of the noncoding genome such as candidate regulatory SNPs, conservation across mammals and its potential effects on regulatory motifs [17]–[19].

## Results

Table 2 compares the basic clinical features of 716 patients with uni and bilateral MD in our series. As we expected, patients with bilateral SNHL had a longer duration of disease (p = 1.5×10^−5^), worse hearing loss at diagnosis (p = 0.003) and worse hearing stage (p = 3×10^−6^), a higher frequency of Tumarkin crises (p = 0.001) and autoimmune disease comorbidities (p = 0.003). However, no differences were observed in the age of onset or frequency of migraine between patients with uni or bilateral SNHL.

**Table 2:** Clinical features of patients with Meniere's disease and uni or bilateral sensorineural hearing loss.

VARIABLES	BILATERAL (n = 168)	UNILATERAL (n = 548)	P-value
**Age of onset, mean ± SD**	46.6±12.5	46.9±12.1	0.743
**Gender (% women)**	60.4	56.6	0.404
**Time course (years), mean ± SD**	11.2±8.7	7.9±6.7	1.5×10^−5^
**Affected ear (%)**		Left (50.6) Right (49.4)	
**Hearing loss at diagnosis, mean ± SD**	53.9±16.6	48.9±17.3	0.003
**Migraine, n (%)**	25 (14.8)	56 (10.2)	0.719
**History autoimmune disease, n (%)**	36 (21.4)	62 (11.3)	0.003
**Smoking, n (%)**	40 (23.8)	134 (29.9)	0.882
**Hearing stage, n (%)**			
**1**	7 (4.2)	58 (12.6)	8.0×10^−6^
**2**	28 (16.6)	116 (21.2)	
**3**	78 (46.4)	260 (47.4)	
**4**	48 (28.5)	69 (12.5)	
**Hearing stage, mean ± SD**	3.01±0.8	2.68±0.9	3.0×10^−6^
**Turmakin crisis, n (%)**	44 (26.1)	77 (14.1)	0.001
**Functional scale, n (%)**			
**1**	28 (16.6)	98 (17.9)	0.964
**2**	46 (27.3)	158 (28.8)	
**3**	36 (21.4)	104 (18.9)	
**4**	25 (14.8)	79 (14.4)	
**5**	16 (9.5)	46 (8.3)	
**6**	3 (1.7)	7 (1.2)	

**Age of onset, time course years and hearing loss at diagnosis were compared by unpaired Student's t test. Qualitative variables were compared by Chi-squared test.**

Principal-component analysis (PCA) showed that cases and controls had similar distributions of the top two eigenvectors in both sets, suggesting a common genetic background for these study subjects (Figure S1 in File S1). We found no evidence of population stratification in the PCA.

No single marker reached a genome-wide significant (p<10^−8^) when all cases and controls were compared. The top ten signals found in patients with MD are shown in Table S1 in File S1. The allelic frequencies of the selected variants in genes *NFKB1, REL, UBE2L3, TNFAIP3* and *TNIP1* in patients and controls are shown in Tables S2, S3, S4, S5, and S6 in File S1. There was no significant difference among patients with uni or bilateral SNHL for any of the SNPs studied (p>0.05). We also stratified patients in two groups according to the presence of uni or bilateral SNHL and compared each group with controls, but none of the allelic variants reached a genome-wide significance. Moreover, none of the selected variants were associated with comorbidities such as autoimmune disorder or migraine.

**Figure 1:**
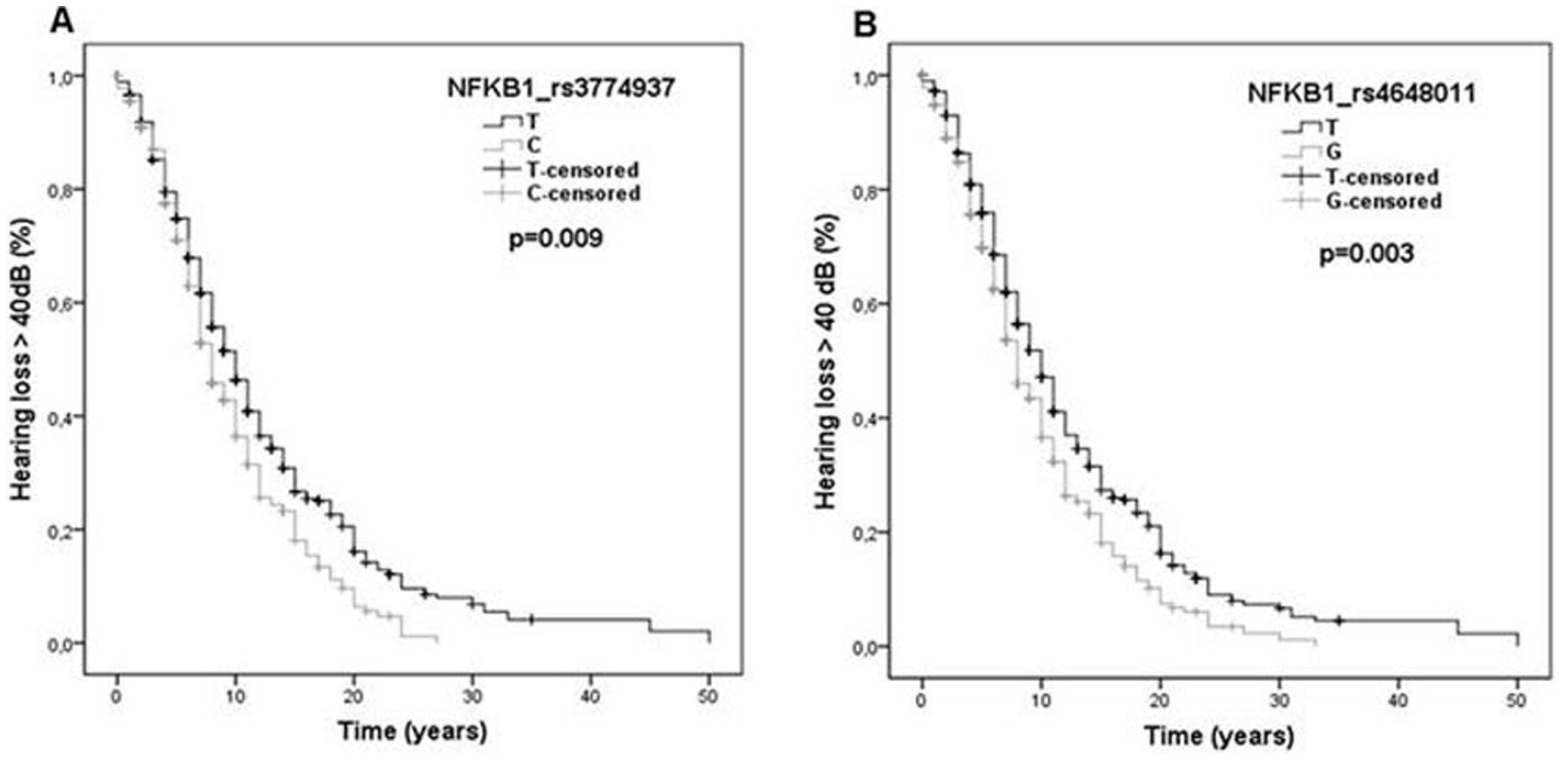
Variants in *NFKB1* gene and hearing outcome in patients with MD were compared by Kaplan-Meier survival curves and the log-rank test. A. Carriers of the C allele in rs3774937 showed a shorter time to reach hearing stage 3 (>40 dB). B. Carriers of the G allele in rs4648011 also reduced in 2 years the mean time to reach hearing stage 3 (log-rank test, p = 0.009 for rs3774937 and p = 0.003 for rs4648011).

**Figure 2:**
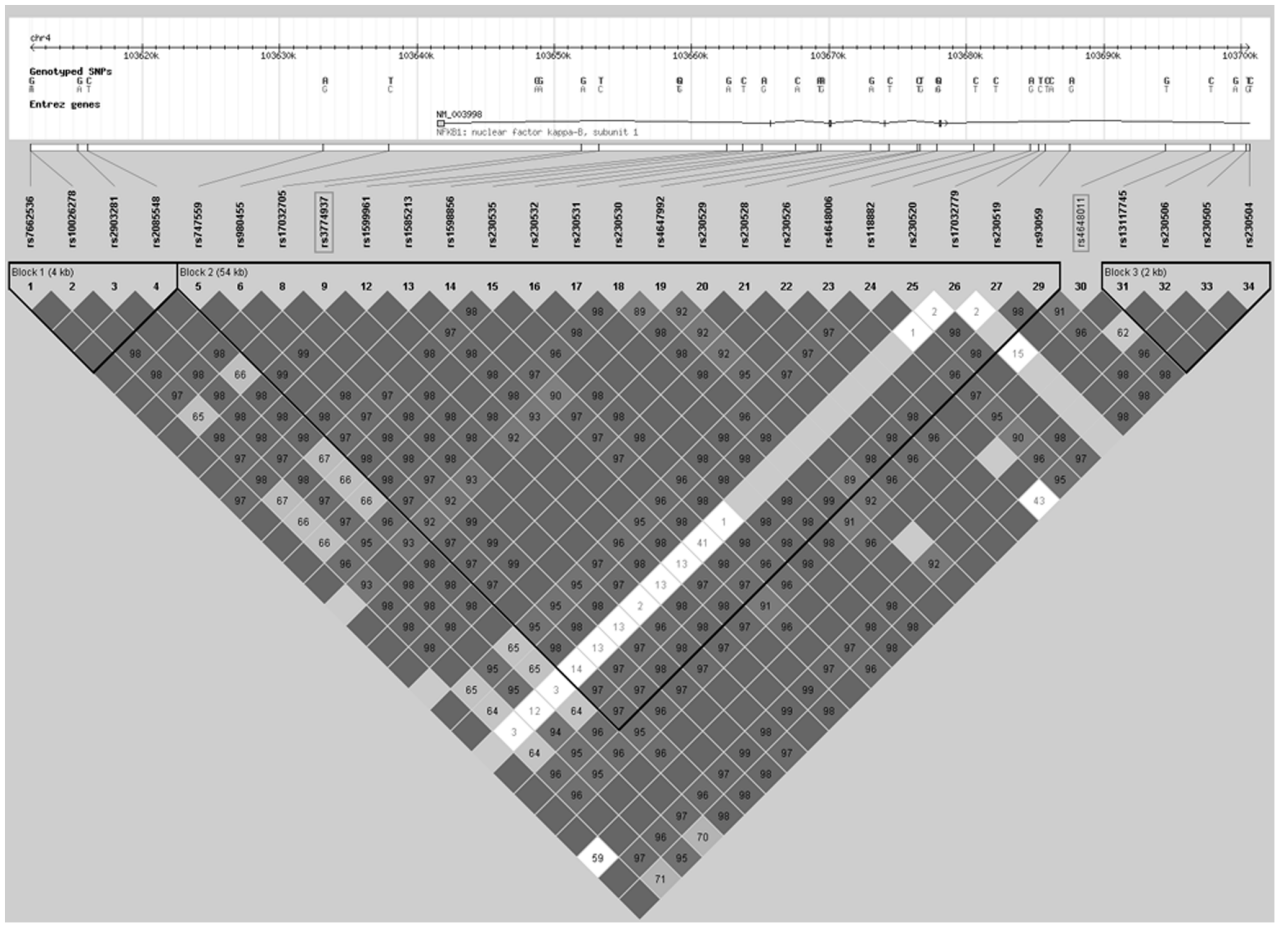
Linkage disequilibrium plot showing the haploblocks with the rs3774937 and rs4648011 (r^2^ = 0.67, D' = 0.95).

We also analyzed the time course of hearing loss in patients with uni or bilateral SNHL for all the functional allelic variants previously selected in the NF-κB pathway. Kaplan-Meier analysis showed that functional allelic variants on *REL, TNFAIP3, REL* and *TNIP1* genes did not influence the auditory prognosis in MD (Table 3). However, two SNP in the *NFKB1* gene (rs3774937 and rs4648011) were associated with a faster hearing loss progression in patients with unilateral SNHL (n = 490). So, patients carrying C allele in rs3774937 and G allele in rs4648011, respectively, reduced in 2 years the mean time to reach hearing stage 3 (>40 dB HL) (log-rank test, corrected p values were p = 0.009 for rs3774937 and p = 0.003 for rs4648011, respectively; Figure 1). So, the median of years to reach hearing stage 3 was 8, 8 or 11 years since the onset of disease for carriers of the genotype CC, CT or TT in rs3774937, respectively (log-rank test, corrected p value was p = 0.018). For rs4648011, we also found that the median time to reach stage 3 was 7, 8 or 11 year since the onset of disease for carriers of the genotype GG, GT or TT, respectively (log-rank test, corrected p value p = 0.018). Remarkably, these variants in the *NFKB1* gene did not influence hearing in patients with bilateral SNHL (p>0.05, 2N = 290). The allelic frequencies of rs3774937-C and rs4648011-G were 0.31 and 0.37, respectively. Both SNPs were validated in all cases by Taqman assays and the correlation coefficient between both methods was 98%. These variants showed high linkage disequilibrium (r^2^ = 0.67, D' = 0.95; Figure 2). The haplotype CG has a frequency of 32% and carriers of this haplotype reached 40 dB 30 months earlier than the rest of the haplotypes carriers (p = 0.002, Table 4). We also compared if carriers of the CG haplotype had a faster hearing loss progression than patients with either rs3774937-C or rs4648011-G alleles, but no additive effect was found.

**Table 3:** Effect of allelic variants in the NF-κB pathway on hearing loss progression in patients with Meniere's disease.

			Long-rank test (p-value)	
GENE	SNV		UNILATERAL	BILATERAL
***TNFAIP3*** ** (intron)**	**rs610604**	**genotype**	0.469	0.431
		**allele**	0.045	0.385
***TNIP1*** ** (intron)**	**rs2233287**	**genotype**	0.806	0.505
		**allele**	0.612	0.521
***TNIP1*** ** (intron)**	**rs1422673**	**genotype**	0.849	0.729
		**allele**	0.303	0.365
***REL*** ** (intron)**	**rs13031237**	**genotype**	0.276	0.053
		**allele**	0.613	0.449
***UBE2L3*** ** (intron)**	**rs5754217**	**genotype**	0.779	0.276
		**allele**	0.992	0.360
***UBE2L3*** ** (intron)**	**rs131654**	**genotype**	0.738	0.208
		**allele**	0.606	0.196
***UBE2L3*** ** (intron)**	**rs181362**	**genotype**	0.779	0.276
		**allele**	0.992	0.360
***NFKB1*** ** (intron)**	**rs3774937**	**genotype (CC)**	**0.018** *	0.160
		**allele (C)**	**0.009** *	0.995
***NFKB1*** ** (intron)**	**rs4648011**	**genotype (GG)**	**0.018** *	0.420
		**allele (G)**	**0.003** *	0.956

Mean time to reach stage 3 (>40 dB) was compared by Kaplan –Meier survival curves and long-rank test.

*corrected p values after Bonferroni's method.

**Table 4:** Effect of rs3774937 (T>C) and rs4648011 (T>G) haplotypes on hearing loss in patients with Meniere's disease.

Time to reach >40 dB (years, mean ± SD)			
HAPLOTYPE (rs3774937, rs4648011)	FREQUENCY (%)	UNILATERAL	BILATERAL
**CG**	33	8±0.48	10±0.90
**GT**	5	8±1.12	12±1.20
**TT**	62	10±0.47	12±0.67

Time to reach hearing >40 dB was compared by survival curves using the Kaplan-Meier method.

Finally, *in silico* analysis of these variants predicted changes in the interaction with the following transcription factors: DMRT1, LUN-1, YY1 for rs3774937 and Foxc1, Zfx for rs4648011.

## Discussion

MD is probably a syndrome including a heterogeneous group of patients with an immune-mediated disease and non-immune mediated mechanisms. The 1995 clinical definition of the AAO-HNS does not discriminate these clinical variants [15].

Our results show that allelic variants in the *NFKB1* gene influence the hearing outcome in patients with unilateral MD. Although these markers are not associated with an increased susceptibility to develop MD, they probably modify the interaction of *NFKB1* with other transcription factors conditioning the inflammatory response in the inner ear. Our study has enough power to detect an association between rs3774937 and rs4648011 and unilateral SNHL patients (>98% for both SNPs). However, the lack of association found in bilateral SNHL patients can be due to the smaller sample size in this cohort, resulting in a lack of power to detect a susceptibility association (power 70% and 66%, respectively). These intronic markers in the NFKB1 gene are located in chromosome 4q24 and they have been strongly associated with primary biliary cirrhosis [45].

Genetic association studies in MD have been limited to case-control studies based on candidate-gene approaches in small series with low power and replication studies have failed to confirm previous associations [20]–[22]. The introduction of technology of genotyping-based arrays after finishing the Human Genome Project [23] and the International HapMap Project [24] have changed the approach for gene discovery in complex diseases to large-scale testing where every gene in the human genome is tested for association with a disease of interest.

The Immunochip project was a collaborative Consortium among 12 immune-mediated disease groups (autoimmune thyroid disease, ankylosing spondylitis, celiac disease, Crohn's disease, IgA deficiency, multiple sclerosis, primary biliary cirrhosis, psoriasis, rheumatoid arthritis, SLE, type 1 diabetes and ulcerative colitis) and the Welcome Trust Case Control Consortium (WTCCC). The result was a high-density array containing 186 distinct susceptibility loci associated with one or more immune-mediated diseases [14]. The ImmunoChip has revealed mainly common variants of modest effect size with odds ratios between 1.04 and 3.99 (mean = 1.29), when excluding the *MHC* region on most of the immune-mediated diseases listed above [25]–[28] and other diseases such as atopic dermatitis [29] or Behcet's disease [30].

We have performed an exploratory genotyping study by using the ImmunoChip including 716 patients and 1628 controls (689 and 1475, respectively after QC), but the clinical heterogeneity of MD anticipates that this sample size is not enough to define the susceptibility loci associated with MD. Previous findings and these results suggest that patients with MD and uni and bilateral SNHL have different genomic background since patients with bilateral SNHL and MD have more frequent comorbid autoimmune diseases [9], [10].

Autoimmunity has been proposed as a mechanism in patients with bilateral SNHL and MD [2] and although preliminary candidate gene studies found that bilateral MD was associated with allelic variants of genes *HLA-DRB1* [31], *PTPN22* [32], these findings have not been formally replicated in the current study including 168 patients with bilateral MD, the largest collection so far.

There are several reasons to explain our findings. The great clinical heterogeneity of MD advances that several thousands of patients will be required to perform a genome-wide association study with enough power to find susceptibility loci with p values <10^−8^. Although those numbers are possible by an international multicenter study for unilateral MD, it is a real challenge to recruit those numbers for bilateral MD.

Different studies suggest the role of innate immune response in the hearing outcome of autoimmune inner ear disease [4], [6] and MD [8]. So, in the present study we have found that two regulatory variants of *NFKB1* gene also influence long-term progression of hearing loss in unilateral MD. Bioinformatics tools predicted that these variants changed the interaction with the following transcription factors: DMRT1, LUN-1, YY1 for rs3774937 and Foxc1, Zfx for rs4648011. Since the NF-κB pathway regulates proinflammatory cytokine production and cell survival and it mediates the duration of the inflammatory response, these variants probably influence gene expression and inflammation in MD. However, further studies with conditioned cells (i.e., lymphoblasts with the homozygous variants) will be necessary to define the effect of these variants in the gene expression profile, since multiple interactions are possible in the NF-κB network and the molecular mechanism remains unknown.

We have recently found that rs11096955 in *TLR10* gene may confer susceptibility to bilateral SNHL in patients with MD [8]. TLRs constitute one of primary defense mechanisms in infections and some noninfectious diseases [33] and inadequate activation of TLRs pathway has been reported in several autoimmune diseases [34]. Since TLRs activates an intracellular signal via MyD88, triggering a complex cascade (IRAK1-IRAK4-TRAF6, TAK1-TAB1-TRAF6-UBC13, IKK complex) that leads to the induction of a large range of proinflammatory genes via the transcription factors NF-κB [35], we have selected functional variants in the genes *NFKB1, REL, UBE2L3, TNFAIP3* and *TNIP1* to evaluate their potential role in the outcome of MD. Overall, we suggest that allelic variants in some genes of the innate immune response such as *TLR10* and *NFKB1* may act as regulatory genes able to modify the clinical progression of hearing in MD. By using an NF-kB reporter mouse, it was demonstrated that the protective action of NF-κB was exerted in connective tissue cells within the spiral ligament. So, in the spiral ligament, type II fibrocytes are activated following systemic inflammatory stress and immune-mediated SNHL in humans may result in part from susceptibility of type II fibrocytes [36]. An abnormal function of type II fibrocytes would likely have a significant impact upon hearing thresholds, since these cells have a critical role in K^+^ ion uptake from perilymph. Moreover, the fact that steroids are potent blockers of NF-κB pathway may explain the observed response to systemic steroids in patients with sudden SNHL or MD.

Our study design has some limitations: the sample size is not enough to detect differences for SNPs with MAF<0.05; however is has been recently demonstrated that the effect of rare variants in autoimmune loci is negligible [37]. We have only compared SNPs included in the ImmunoChip and it is necessary to scan the entire genome in complex diseases. Moreover, the great heterogeneity of the disorder may raise concerns about the cost-effectiveness of this approach. An alternative approach is to select multicase families with MD and combine whole-exome sequencing with segregation analysis to define novel or rare variants in candidate genes. Genomic data fusion including phenotype-and pathway-based analyses may help to decipher the complex genomic architecture of MD.

## Conclusions

Allelic variants rs3774937 and rs4648011 can modify hearing outcome in patients with MD and unilateral SNHL. A patent application number P201430716 has been submitted to the Spanish Patent and Trademark Office.

## Supporting Information

File S1 **Figure S1**, Scatter plot showing the principal component analysis (PCA) in our Spanish samples compared with different populations in HapMap. The eigenvalues for the first three principal components accounted for most of the population substructure in this analysis (77.5%). All individuals who were not clustering with the main cluster (> 3 Standard deviation from cluster center) were excluded from subsequent analysis. Using this method we identified a total of 48 outliers individuals in our case-control cohort. X-axis represents Principal Component 1 (PC1) and Y-axis represents Principal Component 3 (PC3) in our Spanish samples (diamonds), and the main populations in HapMap: CEU, Northern European from Utah (squares), CHB+JPB, Chinese in Beijing+ Japanese in Tokyo (triangles), MEX (crosses), TSI, Tuscans from Italy (asterisks) and YRI, Yoruba in Ibadan, Nigeria (circles). **Table S1**, Minor allelic frequencies of the top 10 ranked signals obtained with the Immunochip in patients with Meniere's diasease. **Table S2**, Minor allelic frequencies of 15 single nucleotide variants in the TNFAIP3 gene in controls and patients with uni and bilateral sensorineural hearing loss. **Table S3**, Minor allelic frequencies of 87 single nucleotide variants in the TNIP1 gene in controls and patients with uni and bilateral sensorineural hearing loss. **Table S4**, Minor allelic frequencies of 16 single nucleotide variants in the REL gene in controls and patients with uni and bilateral sensorineural hearing loss. **Table S5**, Minor allelic frequencies of 34 single nucleotide variants in the UBE2L3 gene in controls and patients with uni and bilateral sensorineural hearing loss. **Table S6**, Minor allelic frequencies of 9 single nucleotide variants in the NFKB1 gene in controls and patients with uni and bilateral sensorineural hearing loss.
(DOCX)
