# Supplementary material for: Intronic Variants in the NFKB1 Gene May Influence Hearing Forecast in Patients with Unilateral Sensorineural Hearing Loss in Meniere's Disease
Source: PLoS One. 2014 Nov 14;9(11):e112171. doi: 10.1371/journal.pone.0112171 (PMC4232390; doi:10.1371/journal.pone.0112171)
Supplement: File S1 — Figure S1, Scatter plot showing the principal component analysis (PCA) in our Spanish samples compared with different populations in HapMap. The eigenvalues for the first three principal components accounted for most of the population substructure in this analysis (77.5%). All individuals who were not clustering with the main cluster (> 3 Standard deviation from cluster center) were excluded from subsequent analysis. Using this method we identified a total of 48 outliers individuals in our case-control cohort. X-axis represents Principal Component 1 (PC1) and Y-axis represents Principal Component 3 (PC3) in our Spanish samples (diamonds), and the main populations in HapMap: CEU, Northern European from Utah (squares), CHB+JPB, Chinese in Beijing+ Japanese in Tokyo (triangles), MEX (crosses), TSI, Tuscans from Italy (asterisks) and YRI, Yoruba in Ibadan, Nigeria (circles). Table S1, Minor allelic frequencies of the top 10 ranked signals obtained with the Immunochip in patients with Meniere's diasease. Table S2, Minor allelic frequencies of 15 single nucleotide variants in the TNFAIP3 gene in controls and patients with uni and bilateral sensorineural hearing loss. Table S3, Minor allelic frequencies of 87 single nucleotide variants in the TNIP1 gene in controls and patients with uni and bilateral sensorineural hearing loss. Table S4, Minor allelic frequencies of 16 single nucleotide variants in the REL gene in controls and patients with uni and bilateral sensorineural hearing loss. Table S5, Minor allelic frequencies of 34 single nucleotide variants in the UBE2L3 gene in controls and patients with uni and bilateral sensorineural hearing loss. Table S6, Minor allelic frequencies of 9 single nucleotide variants in the NFKB1 gene in controls and patients with uni and bilateral sensorineural hearing loss. (DOCX) [file pone.0112171.s001.docx]

**Intronic variants in the** ***NFKB1* gene may influence hearing forecast in patients with unilateral sensorineural hearing loss in Meniere’s disease.**

Sonia Cabrera, Elena Sanchez, Teresa Requena, Manuel Martinez-Bueno, Jesus Benitez, Nicolas Perez, Gabriel Trinidad, Andres Soto-Varela, Sofía Santos-Perez, Eduardo Martin-Sanz, Jesus Fraile, Paz Perez, Marta E Alarcon-Riquelme, Angel Batuecas, Juan M. Espinosa-Sanchez, Ismael Aran, Jose A Lopez-Escamez.

**Supplementary Table S1: Minor allelic frequencies of the top 10 ranked signals obtained with the Immunochip in patients with Meniere’s diasease.**

| SNP | POSITION | CONTROL | A1/A2^a^ | CASES | P | OR(95%CI) |
| --- | --- | --- | --- | --- | --- | --- |
| rs12406128 | chr1:22734220 | 0.335 | A/G | 0.276 | 1,31E-04 | 0.759 (0.659-0.874) |
| rs12023472 | chr1:99921791 | 0.059 | C/G | 0.031 | 1,20E-04 | 0.517 (0.367-0.728) |
| rs10875346 | chr1:101328083 | 0.271 | A/G | 0.216 | 1,02E-04 | 0.739 (0.635-0.861) |
| rs4463689 | chr1:161307723 | 0.091 | A/T | 0.056 | 1,05E-04 | 0.599 (0.461-0.777) |
| rs57995211 | chr1:161340791 | 0.090 | T/A | 0.056 | 1,03E-04 | 0.596 (0.458-0.776) |
| rs11799419 | chr1:172913564 | 0.065 | A/G | 0.037 | 1,38E-04 | 0.545 (0.397-0.748) |
| rs10188379 | chr2:38371476 | 0.189 | C/G | 0.239 | 1,43E-04 | 1.348 (1.155-1.573) |
| rs17031287 | chr2:43920021 | 0.111 | A/T | 0.074 | 1,54E-04 | 0.640 (0.507-0.807) |
| rs1558626 | chr2:102862070 | 0.360 | T/A | 0.300 | 1,20E-04 | 0.763 (0.664-0.875) |
| rs67880537 | chr2:102864106 | 0.357 | G/A | 0.297 | 1,02E-04 | 0.603 (0.662-0.873) |
| rs11191568 | chr10:104886374 | 0.122 | A/G | 0.083 | 1,20E-04 | 0.650 (0.521-0.810) |

**^a^A1/A2: Minor/major allele (based on whole sample)**

**Supplementary Table S2: Minor allelic frequencies of 15 single nucleotide variants in the *TNFAIP3* gene in controls and patients with uni and bilateral sensorineural hearing loss.**

| SNP | POSITION | CONTROL | A1/A2^a^ | UNILATERAL | OR (95% CI) | P | BILATERAL | OR (95% CI) | P |
| --- | --- | --- | --- | --- | --- | --- | --- | --- | --- |
| rs59693083 | Chr6:138186532 | 0.097 | G/A | 0.083 | 0.836 (0.649-1.078) | 0.166 | 0.093 | 0.947 (0.638-1.406) | 0.788 |
| rs5029926 | Chr6:138189515 | 0.097 | G/A | 0.083 | 0.837 (0.650-1.079) | 0.170 | 0.093 | 0.948 (0.639-1.408) | 0.794 |
| rs583522 | Chr6:138189884 | 0.261 | C/T | 0.273 | 1.060 (0.902-1.244) | 0.478 | 0.267 | 1.028 (0.792-1.334) | 0.832 |
| rs5029928 | Chr6:138189942 | 0.096 | T/C | 0.083 | 0.847 (0.657-1.091) | 0.198 | 0.092 | 0.952 (0.641-1.414) | 0.810 |
| rs3757173 | Chr6:138190154 | 0.096 | G/A | 0.083 | 0.847 (0.657-1.091) | 0.198 | 0.093 | 0.959 (0.646-1.424) | 0.836 |
| rs7750604 | Chr6:138190533 | 0.098 | T/C | 0.084 | 0.840 (0.653-1.081) | 0.174 | 0.095 | 0.965 (0.653-1.425) | 0.859 |
| rs5029930 | Chr6:138190684 | 0.097 | C/A | 0.083 | 0.843 (0.654-1.086) | 0.186 | 0.093 | 0.954 (0.643-1.417) | 0.818 |
| rs5029931 | Chr6:138190816 | 0.055 | C/G | 0.044 | 0.790 (0.563-1.108) | 0.171 | 0.058 | 1.064 (0.652-1.737) | 0.802 |
| rs5029933 | Chr6:138192062 | 0.055 | G/A | 0.044 | 0.782 (0.558-1.097) | 0.153 | 0.059 | 1.065 (0.652-1.738) | 0.800 |
| rs719149 | Chr6:138192745 | 0.096 | A/G | 0.083 | 0.847 (0.657-1.092) | 0.201 | 0.089 | 0.918 (0.615-1.371) | 0.678 |
| rs719150 | Chr6:138192761 | 0.097 | G/A | 0.083 | 0.844 (0.655-1.088) | 0.190 | 0.092 | 0.949 (0.639-1.41) | 0.798 |
| rs629953 | Chr6:138195041 | 0.366 | A/G | 0.354 | 0.951 (0.819-1.104) | 0.512 | 0.358 | 0.964 (0.759-1.225) | 0.767 |
| rs643177 | Chr6:138195693 | 0.270 | T/C | 0.271 | 1.005 (0.855-1.180) | 0.955 | 0.264 | 0.969 (0.746-1.258) | 0.813 |
| rs661561 | Chr6:138197331 | 0.365 | A/C | 0.356 | 0.960 (0.827-1.114) | 0.594 | 0.356 | 0.960 (0.755-1222) | 0.744 |
| rs610604 | Chr6:138199417 | 0.332 | G/T | 0.328 | 0.984 (0.846-1.146) | 0.841 | 0.332 | 1.000 (0.783-1.277) | 0.997 |

**Supplementary Table S3: Minor allelic frequencies of 87 single nucleotide variants in the *TNIP1* gene in controls and patients with uni and bilateral sensorineural hearing loss.**

| SNP | POSITION | CONTROL | A1 /A2 | UNILATERAL | OR (95% CI) | P | BILATERAL | OR (95% CI) | P |
| --- | --- | --- | --- | --- | --- | --- | --- | --- | --- |
| rs736775 | Chr5:150409348 | 0.399 | T/C | 0.390 | 0.961 (0.830-1.112) | 0.594 | 0.398 | 0.993 (0.786-1.256) | 0.958 |
| rs2277940 | Chr5:150409477 | 0.076 | C/T | 0.071 | 0.933 (0.709-1.228) | 0.622 | 0.068 | 0.895 (0.568-1.410) | 0.634 |
| rs8177834 | Chr5:150409989 | 0.099 | T/C | 0.098 | 0.991 (0.781-1.258) | 0.943 | 0.080 | 0.788 (0.519-1.199) | 0.265 |
| rs3924 | Chr5:150410135 | 0.402 | G/A | 0.401 | 0.998 (0.863-1.155) | 0.980 | 0.410 | 1.036 (0.818-1.313) | 0.767 |
| rs2233311 | Chr5:150410219 | 0.101 | T/G | 0.098 | 0.972 (0.766-1.234) | 0.821 | 0.080 | 0.774 (0.509-1.176) | 0.229 |
| rs10463311 | Chr5:150410835 | 0.295 | G/A | 0.300 | 1.025 (0.877-1.197) | 0.757 | 0.321 | 1.128 (0.881-1.444) | 0.336 |
| rs10463312 | Chr5:150410894 | 0.401 | T/C | 0.400 | 0.993 (0.858-1.149) | 0.928 | 0.407 | 1.024 (0.810-1.293) | 0.845 |
| rs3763009 | Chr5:150412140 | 0.315 | A/G | 0.357 | 1.207 (1.039-1.402) | 0.013 | 0.299 | 0.928 (0.723-1.193) | 0.561 |
| rs72790107 | Chr5:150412836 | 0.100 | T/C | 0.098 | 0.985 (0.776-1.250) | 0.904 | 0.077 | 0.756 (0.494-1.158) | 0.197 |
| rs2233305 | Chr5:150413406 | 0.214 | A/C | 0.213 | 0.993 (0.835-1.182) | 0.942 | 0.248 | 1.211 (0.926-1.583) | 0.159 |
| rs60602178 | Chr5:150413528 | 0.078 | G/A | 0.074 | 0.946 (0.723-1.240) | 0.690 | 0.077 | 0.979 (0.637-1.505) | 0.923 |
| rs11747926 | Chr5:150414388 | 0.191 | A/G | 0.182 | 0.941 (0.783-1.131) | 0.520 | 0.206 | 1.103 (0.830-1.466) | 0.498 |
| rs11748040 | Chr5:150414945 | 0.124 | A/G | 0.122 | 0.986 (0.794-1.226) | 0.905 | 0.140 | 1.156 (0.828-1.614) | 0.393 |
| rs11748041 | Chr5:150414960 | 0.204 | A/C | 0.186 | 0.893 (0.744-1.071) | 0.221 | 0.206 | 1.017 (0.766-1.350) | 0.907 |
| rs2287720 | Chr5:150415338 | 0.398 | C/T | 0.398 | 1.001 (0.864-1.159) | 0.985 | 0.402 | 1.018 (0.803-1.289) | 0.883 |
| rs72790109 | Chr5:150418221 | 0.098 | A/G | 0.095 | 0.968 (0.761-1.232) | 0.794 | 0.080 | 0.802 (0.527-1.219) | 0.301 |
| rs62382333 | Chr5:150418959 | 0.076 | C/T | 0.072 | 0.938 (0.714-1.232) | 0.646 | 0.067 | 0.875 (0.556-1.378) | 0.565 |
| rs56661379 | Chr5:150419962 | 0.077 | C/T | 0.072 | 0.930 (0.708-1.222) | 0.604 | 0.070 | 0.910 (0.583-1.421) | 0.681 |
| rs72790110 | Chr5:150420038 | 0.099 | A/G | 0.098 | 0.989 (0.779-1.255) | 0.928 | 0.080 | 0.787 (0.517-1.196) | 0.261 |
| rs9324672 | Chr5:150420056 | 0.105 | A/C | 0.111 | 1.066 (0.849-1.338) | 0.582 | 0.089 | 0.834 (0.559-1.244) | 0.374 |

| SNP | POSITION | CONTROL | A1 /A2 | UNILATERAL | OR (95% CI) | P | BILATERAL | OR (95% CI) | P |
| --- | --- | --- | --- | --- | --- | --- | --- | --- | --- |
| rs3805434 | Chr5:150420339 | 0.173 | C/G | 0.164 | 0.938 (0.775-1.137) | 0.517 | 0.182 | 1.062 (0.788-1.431) | 0.691 |
| rs4958876 | Chr5:150422765 | 0.131 | A/C | 0.131 | 1.001 (0.810-1.236) | 0.995 | 0.158 | 1.248 (0.908-1.715) | 0.171 |
| rs10051105 | Chr5:150423459 | 0.074 | G/A | 0.067 | 0.901 (0.681-1.193) | 0.468 | 0.070 | 0.951 (0.609-1.486) | 0.826 |
| rs62382335 | Chr5:150425032 | 0.080 | T/A | 0.077 | 0.962 (0.738-1.256) | 0.780 | 0.070 | 0.878 (0.563-1.370) | 0.566 |
| rs2233299 | Chr5:150425467 | 0.282 | A/G | 0.240 | 0.806 (0.684-0.951) | 0.010 | 0.285 | 1.018 (0.789-1.314) | 0.889 |
| rs2161359 | Chr5:150426534 | 0.322 | A/G | 0.352 | 1.146 (0.986-1.332) | 0.075 | 0.296 | 0.885 (0.689-1.138) | 0.343 |
| rs11747787 | Chr5:150426868 | 0.482 | A/C | 0.439 | 0.840 (0.728-0.970) | 0.017 | 0.478 | 0.983 (0.781-1.239) | 0.889 |
| rs7734456 | Chr5:150427172 | 0.392 | C/G | 0.429 | 1.167 (1.010-1.348) | 0.036 | 0.366 | 0.896 (0.706-1.138) | 0.370 |
| rs12655899 | Chr5:150428157 | 0.079 | A/G | 0.077 | 0.971 (0.745-1.267) | 0.831 | 0.074 | 0.927 (0.599-1.436) | 0.736 |
| rs4292439 | Chr5:150428195 | 0.204 | C/T | 0.199 | 0.964 (0.807-1.153) | 0.693 | 0.231 | 1.170 (0.890-1.537) | 0.260 |
| rs13153275 | Chr5:150428399 | 0.282 | C/G | 0.243 | 0.816 (0.692-0.962) | 0.015 | 0.284 | 1.005 (0.779-1.297) | 0.968 |
| rs72790117 | Chr5:150428746 | 0.099 | A/G | 0.095 | 0.958 (0.753-1.220) | 0.732 | 0.080 | 0.788 (0.519-1.199) | 0.265 |
| rs2233297 | Chr5:150429240 | 0.071 | C/T | 0.067 | 0.937 (0.707-1.242) | 0.651 | 0.070 | 0.989 (0.632-1.546) | 0.961 |
| rs2233294 | Chr5:150429563 | 0.222 | C/A | 0.190 | 0.820 (0.685-0.980) | 0.029 | 0.220 | 0.987 (0.748-1.304) | 0.931 |
| rs7713567 | Chr5:150430955 | 0.324 | T/C | 0.298 | 0.885 (0.758-1.034) | 0.123 | 0.347 | 1.110 (0.871-1.414) | 0.397 |
| rs888989 | Chr5:150431030 | 0.192 | C/T | 0.177 | 0.903 (0.751-1.088) | 0.284 | 0.169 | 0.858 (0.633-1.164) | 0.326 |
| rs2112635 | Chr5:150432153 | 0.319 | C/T | 0.298 | 0.906 (0.776-1.058) | 0.213 | 0.306 | 0.940 (0.732-1.208) | 0.630 |
| rs871269 | Chr5:150432388 | 0.316 | T/C | 0.336 | 1.096 (0.941-1.276) | 0.235 | 0.330 | 1.067 (0.835-1.362) | 0.604 |
| rs17111695 | Chr5:150432446 | 0.124 | C/T | 0.126 | 1.017 (0.820-1.261) | 0.874 | 0.124 | 0.996 (0.703-1.413) | 0.985 |
| rs34294852 | Chr5:150433447 | 0.223 | C/T | 0.222 | 0.996 (0.839-1.183) | 0.964 | 0.248 | 1.150 (0.877-1.507) | 0.310 |
| rs6895271 | Chr5:150434421 | 0.220 | T/C | 0.220 | 0.999 (0.841-1.187) | 0.993 | 0.246 | 1.161 (0.888-1.516) | 0.274 |
| rs4958879 | Chr5:150434422 | 0.453 | G/A | 0.441 | 0.952 (0.825-1.099) | 0.505 | 0.450 | 0.989 (0.785-1.246) | 0.925 |
| rs3792794 | Chr5:150434722 | 0.068 | T/C | 0.071 | 1.052 (0.796-1.389) | 0.722 | 0.055 | 0.804 (0.489-1.322) | 0.389 |
| rs6579837 | Chr5:150434894 | 0.102 | T/G | 0.104 | 1.029 (0.814-1.299) | 0.810 | 0.101 | 0.997 (0.682-1.458) | 0.990 |

| SNP | POSITION | CONTROL | A1/A2 | UNILATERAL | OR (95% CI) | P | BILATERAL | OR (95% CI) | P |
| --- | --- | --- | --- | --- | --- | --- | --- | --- | --- |
| rs3805433 | Chr5:150435480 | 0.251 | G/C | 0.239 | 0.933 (0.790-1.103) | 0.419 | 0.274 | 1.125 (0.869-1.456) | 0.371 |
| rs62382336 | Chr5:150435525 | 0.064 | A/G | 0.061 | 0.951 (0.709-1.277) | 0.740 | 0.081 | 1.281 (0.835-1.963) | 0.254 |
| rs12516176 | Chr5:150435645 | 0.251 | C/T | 0.239 | 0.935 (0.792-1.105) | 0.432 | 0.273 | 1.119 (0.863-1.450) | 0.394 |
| rs12518386 | Chr5:150438085 | 0.220 | A/G | 0.220 | 0.999 (0.841-1.187) | 0.998 | 0.246 | 1.158 (0.885-1.515) | 0.284 |
| rs4958435 | Chr5:150438284 | 0.449 | T/G | 0.433 | 0.935 (0.810-1.080) | 0.362 | 0.432 | 0.930 (0.737-1.173) | 0.540 |
| rs4958880 | Chr5:150438477 | 0.195 | A/C | 0.195 | 0.995 (0.831-1.191) | 0.958 | 0.158 | 0.772 (0.565-1.056) | 0.105 |
| rs1422673 | Chr5:150438988 | 0.196 | T/C | 0.194 | 0.987 (0.824-1.182) | 0.887 | 0.160 | 0.781 (0.572-1.066) | 0.118 |
| rs2042234 | Chr5:150439131 | 0.096 | G/A | 0.095 | 0.987 (0.774-1.258) | 0.917 | 0.078 | 0.799 (0.522-1.225) | 0.303 |
| rs3805431 | Chr5:150439539 | 0.067 | A/G | 0.070 | 1.056 (0.798-1.398) | 0.701 | 0.055 | 0.818 (0.497-1.345) | 0.427 |
| rs2233287 | Chr5:150440097 | 0.098 | A/G | 0.096 | 0.977 (0.767-1.244) | 0.850 | 0.083 | 0.830 (0.549-1.254) | 0.376 |
| rs17728260 | Chr5:150443005 | 0.078 | G/A | 0.087 | 1.135 (0.878-1.466) | 0.332 | 0.068 | 0.872 (0.554-1.373) | 0.555 |
| rs17111708 | Chr5:150443507 | 0.097 | A/G | 0.098 | 1.006 (0.791-1.279) | 0.958 | 0.086 | 0.881 (0.587-1.324) | 0.543 |
| rs73272818 | Chr5:150444843 | 0.098 | C/T | 0.099 | 1.005 (0.791-1.276) | 0.967 | 0.086 | 0.865 (0.576-1.298) | 0.483 |
| rs10057690 | Chr5:150445215 | 0.101 | C/T | 0.105 | 1.046 (0.828-1.320) | 0.708 | 0.089 | 0.869 (0.583-1.298) | 0.494 |
| rs1559127 | Chr5:150446753 | 0.129 | C/T | 0.124 | 0.958 (0.773-1.189) | 0.700 | 0.121 | 0.928 (0.653-1.321) | 0.681 |
| rs6880110 | Chr5:150447090 | 0.158 | G/A | 0.144 | 0.891 (0.729-1.090) | 0.263 | 0.138 | 0.854 (0.614-1.188) | 0.349 |
| rs4958881 | Chr5:150450236 | 0.127 | C/T | 0.116 | 0.904 (0.725-1.126) | 0.368 | 0.112 | 0.867 (0.603-1.248) | 0.443 |
| rs75805068 | Chr5:150451075 | 0.099 | T/A | 0.097 | 0.985 (0.775-1.251) | 0.901 | 0.093 | 0.933 (0.628-1.385) | 0.730 |
| rs3792785 | Chr5:150451650 | 0.105 | C/T | 0.101 | 0.963 (0.762-1.219) | 0.758 | 0.092 | 0.868 (0.585-1.287) | 0.481 |
| rs13160369 | Chr5:150452196 | 0.164 | C/G | 0.141 | 0.834 (0.681-1.021) | 0.078 | 0.142 | 0.839 (0.605-1.165) | 0.295 |
| rs6869605 | Chr5:150452866 | 0.136 | C/A | 0.123 | 0.890 (0.718-1.103) | 0.288 | 0.126 | 0.915 (0.648-1.291) | 0.614 |

| SNP | POSITION | CONTROL | A1 / A2 | UNILATERAL | OR (95% CI) | P | BILATERAL | OR (95% CI) | P |  |
| --- | --- | --- | --- | --- | --- | --- | --- | --- | --- | --- |
| rs17111727 | Chr5:150455442 | 0.064 | G/A | 0.047 | 0.730 (0.529-1.008) | 0.055 | 0.058 | 0.903 (0.555-1.469) | 0.682 |  |
| rs3792784 | Chr5:150455672 | 0.106 | G/A | 0.100 | 0.939 (0.741-1.190) | 0.604 | 0.092 | 0.860 (0.580-1.276) | 0.455 |  |
| rs3792782 | Chr5:150456677 | 0.464 | T/C | 0.451 | 0.946 (0.820-1.092) | 0.450 | 0.459 | 0.980 (0.778-1.235) | 0.867 |  |
| rs6889239 | Chr5:150457771 | 0.249 | C/T | 0.243 | 0.967 (0.819-1.141) | 0.692 | 0.209 | 0.795 (0.599-1.054) | 0.110 | |
| rs4958438 | Chr5:150461069 | 0.088 | G/A | 0.101 | 1.160 (0.913-1.473) | 0.224 | 0.080 | 0.893 (0.586-1.360) | 0.598 |  |
| rs28372811 | Chr5:150461285 | 0.081 | G/A | 0.098 | 1.233 (0.964-1.575) | 0.093 | 0.067 | 0.826 (0.525-1.299) | 0.408 |  |
| rs13168551 | Chr5:150462638 | 0.396 | G/A | 0.382 | 0.943 (0.815-1.093) | 0.438 | 0.382 | 0.942 (0.743-1.194) | 0.622 |  |
| rs62383767 | Chr5:150462705 | 0.091 | A/G | 0.086 | 0.939 (0.729-1.209) | 0.626 | 0.055 | 0.586 (0.358-0.959) | 0.031 |  |
| rs7702954 | Chr5:150463695 | 0.502 | G/C | 0.479 | 0.910 (0.789-1.051) | 0.200 | 0.481 | 0.938 (0.746-1.181) | 0.590 |  |
| rs115682037 | Chr5:150464017 | 0.070 | G/A | 0.071 | 1.016 (0.770-1.341) | 0.908 | 0.077 | 1.110 (0.720-1.710) | 0.636 |  |
| rs75851973 | Chr5:150464579 | 0.065 | G/A | 0.055 | 0.839 (0.619-1.138) | 0.259 | 0.068 | 1.049 (0.664-1.657) | 0.836 |  |
| rs76956521 | Chr5:150464641 | 0.064 | C/A | 0.055 | 0.859 (0.633-1.166) | 0.328 | 0.071 | 1.119 (0.713-1.753) | 0.624 |  |
| rs4958883 | Chr5:150465247 | 0.419 | A/G | 0.416 | 0.987 (0.854-1.141) | 0.860 | 0.437 | 1.077 (0.853-1.358) | 0.533 |  |
| rs918499 | Chr5:150465555 | 0.419 | A/G | 0.415 | 0.983 (0.851-1.137) | 0.824 | 0.434 | 1.063 (0.842-1.341) | 0.606 |  |
| rs2017638 | Chr5:150465603 | 0.414 | G/A | 0.417 | 1.014 (0.877-1.172) | 0.851 | 0.432 | 1.076 (0.853-1.357) | 0.537 |  |
| rs3792780 | Chr5:150466193 | 0.360 | C/A | 0.365 | 1.019 (0.878-1.181) | 0.808 | 0.385 | 1.110 (0.875-1.406) | 0.388 |  |
| rs3792779 | Chr5:150466385 | 0.364 | C/A | 0.364 | 0.996 (0.859-1.156) | 0.964 | 0.385 | 1.091 (0.860-1.382) | 0.472 |  |
| rs2287724 | Chr5:150466498 | 0.360 | G/A | 0.365 | 1.025 (0.884-1.189) | 0.741 | 0.385 | 1.116 (0.881-1.413) | 0.362 |  |
| rs2287725 | Chr5:150466504 | 0.431 | G/A | 0.420 | 0.957 (0.828-1.105) | 0.549 | 0.453 | 1.094 (0.868-1.378) | 0.443 |  |
| rs2233279 | Chr5:150467170 | 0.360 | C/T | 0.363 | 1.011 (0.871-1.173) | 0.884 | 0.385 | 1.111 (0.876-1.408) | 0.383 |  |
| rs2233278 | Chr5:150467189 | 0.065 | C/G | 0.051 | 0.777 (0.568-1.063) | 0.113 | 0.07 | 1.084 (0.692-1.697) | 0.724 |  |
| rs2233274 | Chr5:150467475 | 0.364 | C/T | 0.362 | 0.991 (0.854-1.150) | 0.908 | 0.382 | 1.082 (0.854-1.371) | 0.512 |  |
| rs75970310 | Chr5:150468123 | 0.140 | G/A | 0.138 | 0.979 (0.797-1.203) | 0.841 | 0.188 | 1.419 (1.054-1.911) | 0.020 |  |

**Supplementary Table S4: Minor allelic frequencies of 16 single nucleotide variants in the *REL* gene in controls and patients with uni and bilateral sensorineural hearing loss.**

| SNP | POSITION | CONTROL | A1 /A2 | UNILATERAL | OR (95% CI) | P | BILATERAL | OR (95% CI) | P |
| --- | --- | --- | --- | --- | --- | --- | --- | --- | --- |
| rs67574266 | Chr2:61108829 | 0.382 | A/G | 0.383 | 1.004 (0.866-1.163) | 0.957 | 0.363 | 0.922 (0.726-1.172) | 0.509 |
| rs842650 | Chr2:61108996 | 0.097 | A/T | 0.104 | 1.075 (0.849-1.360) | 0.549 | 0.108 | 1.120 (0.772-1.623) | 0.550 |
| rs6545835 | Chr2:61112552 | 0.255 | T/G | 0.243 | 0.934 (0.792-1.103) | 0.420 | 0.282 | 1.146 (0.886-1.481) | 0.297 |
| rs77787761 | Chr2:61116257 | 0.096 | G/A | 0.105 | 1.111 (0.879-1.404) | 0.377 | 0.108 | 1.139 (0.785-1.651) | 0.492 |
| rs842648 | Chr2:61116481 | 0.095 | C/T | 0.105 | 1.115 (0.881-1.409) | 0.363 | 0.108 | 1.142 (0.787-1.657) | 0.482 |
| rs12713428 | Chr2:61118113 | 0.257 | C/A | 0.244 | 0.930 (0.789-1.098) | 0.395 | 0.277 | 1.109 (0.857-1.433) | 0.431 |
| rs34493725 | Chr2:61118309 | 0.243 | T/C | 0.222 | 0.888 (0.749-1.053) | 0.172 | 0.212 | 0.837 (0.632-1.109) | 0.214 |
| rs10185028 | Chr2:61118739 | 0.255 | G/A | 0.245 | 0.945 (0.801-1.115) | 0.504 | 0.277 | 1.120 (0.866-1.448) | 0.387 |
| rs10193964 | Chr2:61126664 | 0.264 | G/A | 0.256 | 0.962 (0.817-1.132) | 0.640 | 0.284 | 1.104 (0.855-1.424) | 0.448 |
| rs6707682 | Chr2:61128268 | 0.268 | A/G | 0.242 | 0.871 (0.739-1.028) | 0.103 | 0.284 | 1.082 (0.838-1.396) | 0.545 |
| rs9752570 | Chr2:61132029 | 0.263 | T/A | 0.242 | 0.896 (0.759-1.057) | 0.192 | 0.280 | 1.092 (0.845-1.411) | 0.499 |
| rs7604989 | Chr2:61133793 | 0.262 | G/A | 0.249 | 0.929 (0.788-1.095) | 0.382 | 0.284 | 1.112 (0.861-1.435) | 0.416 |
| rs72807480 | Chr2:61134764 | 0.260 | C/A | 0.249 | 0.940 (0.798-1.108) | 0.464 | 0.287 | 1.142 (0.885-1.473) | 0.306 |
| rs13031237 | Chr2:61136129 | 0.378 | T/G | 0.379 | 1.004 (0.866-1.163) | 0.959 | 0.357 | 0.912 (0.717-1.160) | 0.452 |
| rs34683507 | Chr2:61151333 | 0.212 | T/C | 0.203 | 0.944 (0.791-1.127) | 0.527 | 0.203 | 0.945 (0.711-1.257) | 0.701 |
| rs36047498 | Chr2:61151661 | 0.123 | A/T | 0.125 | 1.015 (0.818-1.258) | 0.894 | 0.117 | 0.939 (0.658-1.341) | 0.731 |

**Supplementary Table S5: Minor allelic frequencies of 34 single nucleotide variants in the *UBE2L3* gene in controls and patients with uni and bilateral sensorineural hearing loss.**

| SNP | POSITION | CONTROL | A1 / A2 | UNILATERAL | OR (95% CI) | P | BILATERAL | OR (95% CI) | P |
| --- | --- | --- | --- | --- | --- | --- | --- | --- | --- |
| rs131654 | Chr22:21917190 | 0.289 | G/T | 0.295 | 1.027 (0.878-1.201) | 0.737 | 0.243 | 0.791 (0.606-1.032) | 0.083 |
| rs131665 | Chr22:21920903 | 0.203 | C/T | 0.203 | 1.000 (0.837-1.194) | 0.999 | 0.231 | 1.179 (0.895-1.552) | 0.241 |
| rs140489 | Chr22:21921294 | 0.217 | T/C | 0.213 | 0.978 (0.822-1.164) | 0.804 | 0.240 | 1.144 (0.873-1.498) | 0.327 |
| rs2266959 | Chr22:21922904 | 0.199 | T/G | 0.202 | 1.021 (0.854-1.219) | 0.820 | 0.220 | 1.138 (0.861-1.504) | 0.362 |
| rs140492 | Chr22:21923144 | 0.210 | C/A | 0.212 | 1.017 (0.854-1.210) | 0.851 | 0.240 | 1.193 (0.911-1.562) | 0.199 |
| rs2256609 | Chr22:21925017 | 0.203 | G/A | 0.202 | 0.998 (0.835-1.192) | 0.984 | 0.220 | 1.109 (0.839-1.466) | 0.467 |
| rs79858848 | Chr22:21926884 | 0.092 | G/A | 0.082 | 0.878 (0.680-1.134) | 0.318 | 0.095 | 1.035 (0.700-1.530) | 0.862 |
| rs140498 | Chr22:21927064 | 0.205 | A/G | 0.204 | 0.995 (0.834-1.188) | 0.963 | 0.232 | 1.176 (0.894-1546) | 0.244 |
| rs140499 | Chr22:21927231 | 0.205 | T/C | 0.203 | 0.993 (0.832-1.185) | 0.939 | 0.228 | 1.148 (0.872-1.510) | 0.325 |
| rs2266961 | Chr22:21928597 | 0.198 | C/G | 0.203 | 1.031 (0.863-1.231) | 0.733 | 0.220 | 1.142 (0.864-1.508) | 0.351 |
| rs181360 | Chr22:21928916 | 0.204 | C/A | 0.203 | 0.994 (0.833-1.186) | 0.947 | 0.228 | 1.149 (0.873-1.511) | 0.321 |
| rs181362 | Chr22:21932068 | 0.210 | T/C | 0.213 | 1.018 (0.855-1.211) | 0.841 | 0.240 | 1.190 (0.908-1.559) | 0.205 |
| rs181363 | Chr22:21932264 | 0.210 | G/A | 0.212 | 1.014 (0.852-1.207) | 0.874 | 0.240 | 1.190 (0.908-1.558) | 0.205 |
| rs181366 | Chr22:21933780 | 0.218 | A/G | 0.203 | 0.915 (0.768-1.092) | 0.327 | 0.228 | 1.058 (0.804-1.392) | 0.685 |
| rs5754217 | Chr22:21939675 | 0.217 | T/G | 0.213 | 0.976 (0.820-1.162) | 0.786 | 0.240 | 1.142 (0.871-1.495) | 0.335 |
| rs12484550 | Chr22:21941915 | 0.198 | T/C | 0.203 | 1.033 (0.864-1.233) | 0.722 | 0.217 | 1.123 (0.848-1.485) | 0.418 |
| rs5998599 | Chr22:21941981 | 0.202 | G/A | 0.203 | 1.011 (0.847-1.206) | 0.904 | 0.219 | 1.107 (0.838-1.463) | 0.471 |

| SNP | POSITION | CONTROL | A1/A2 | UNILATERAL | OR(95%CI) | P | BILATERAL | OR(95% CI) | P |
| --- | --- | --- | --- | --- | --- | --- | --- | --- | --- |
| rs9621715 | Chr22:21942007 | 0.198 | A/G | 0.203 | 1.030 (0.863-1.230) | 0.740 | 0.217 | 1.120 (0.846-1.482) | 0.426 |
| rs2283789 | Chr22:21944478 | 0.208 | G/T | 0.204 | 0.978 (0.819-1.167) | 0.805 | 0.226 | 1.115 (0.846-1.470) | 0.437 |
| rs5998619 | Chr22:21945851 | 0.203 | A/G | 0.204 | 1.009 (0.845-1.204) | 0.921 | 0.220 | 1.110 (0.840-1.466) | 0.461 |
| rs73166630 | Chr22:21945978 | 0.201 | A/G | 0.203 | 1.013 (0.849-1.210) | 0.882 | 0.225 | 1.154 (0.875-1.520) | 0.309 |
| rs73166632 | Chr22:21946173 | 0.203 | G/A | 0.204 | 1.004 (0.841-1.198) | 0.966 | 0.226 | 1.145 (0.868-1.508) | 0.336 |
| rs2070512 | Chr22:21949411 | 0.210 | C/A | 0.212 | 1.017 (0.854-1.210) | 0.851 | 0.240 | 1.193 (0.911-1.562) | 0.199 |
| rs2283790 | Chr22:21956653 | 0.204 | G/A | 0.203 | 0.999 (0.837-1.192) | 0.991 | 0.231 | 1.175 (0.893-1.544) | 0.247 |
| rs11089629 | Chr22:21958872 | 0.208 | G/T | 0.213 | 1.030 (0.865-1.226) | 0.742 | 0.242 | 1.211 (0.924-1.587) | 0.164 |
| rs4821108 | Chr22:21959038 | 0.213 | C/G | 0.202 | 0.936 (0.784-1.117) | 0.465 | 0.225 | 1.070 (0.812-1.409) | 0.632 |
| rs4821112 | Chr22:21964761 | 0.205 | A/G | 0.204 | 0.991 (0.831-1.183) | 0.922 | 0.229 | 1.151 (0.874-1.514) | 0.316 |
| rs5754352 | Chr22:21964951 | 0.207 | T/C | 0.203 | 0.981 (0.822-1.171) | 0.834 | 0.226 | 1.123 (0.852-1.480) | 0.408 |
| rs5998672 | Chr22:21966442 | 0.211 | A/G | 0.212 | 1.006 (0.845-1.198) | 0.944 | 0.242 | 1.190 (0.908-1.559) | 0.205 |
| rs738127 | Chr22:21968221 | 0.205 | A/G | 0.203 | 0.991 (0.831-1.184) | 0.928 | 0.228 | 1.146 (0.871-1.508) | 0.329 |
| rs738129 | Chr22:21971041 | 0.212 | T/C | 0.215 | 1.016 (0.854-1.208) | 0.857 | 0.240 | 1.174 (0.896-1.538) | 0.242 |
| rs4821116 | Chr22:21973319 | 0.199 | T/C | 0.203 | 1.027 (0.860-1.226) | 0.768 | 0.216 | 1.108 (0.837-1.465) | 0.473 |
| rs7444 | Chr22:21976934 | 0.209 | C/T | 0.203 | 0.960 (0.803-1.148) | 0.656 | 0.232 | 1.143 (0.868-1.506) | 0.339 |
| rs7445 | Chr22:21977047 | 0.207 | T/C | 0.205 | 0.987 (0.828-1.177) | 0.887 | 0.231 | 1.150 (0.875-1.511) | 0.315 |

**Supplementary Table S6: Minor allelic frequencies of 9 single nucleotide variants in the *NFKB1* gene in controls and patients with uni and bilateral sensorineural hearing loss.**

| SNP | POSITION | CONTROL | A1/A2 | UNILATERAL | OR(95%CI) | P | BILATERAL | OR(95% CI) | P |
| --- | --- | --- | --- | --- | --- | --- | --- | --- | --- |
| rs6533014 | Chr4:103349740 | 0.491 | G/A | 0.476 | 0.942 (0.817-1.088) | 0.418 | 0.453 | 0.859 (0.681-1.083) | 0.197 |
| rs6846971 | Chr4:103359744 | 0.214 | T/C | 0.218 | 1.024 (0.860-1.217) | 0.791 | 0.214 | 1.000 (0.755-1.325) | 0.998 |
| rs17032705 | Chr4:103432974 | 0.361 | A/G | 0.370 | 1.041 (0.897-1.207) | 0.594 | 0.315 | 0.814 (0.635-1.043) | 0.103 |
| rs3774937 | Chr4:103434253 | 0.323 | C/T | 0.317 | 0.970 (0.832-1.131) | 0.700 | 0.260 | 0.736 (0.567-0.956) | 0.021 |
| rs4648011 | Chr4:103475444 | 0.363 | G/T | 0.373 | 1.043 (0.898-1.210) | 0.581 | 0.335 | 0.882 (0.691-1.126) | 0.315 |
| rs13117745 | Chr4:103478703 | 0.164 | T/C | 0.152 | 0.918 (0.754-1.118) | 0.397 | 0.192 | 1.213 (0.904-1.628) | 0.196 |
| rs4648090 | Chr4:103527068 | 0.167 | A/G | 0.151 | 0.888 (0.730-1.082) | 0.239 | 0.137 | 0.792 (0.568-1.105) | 0.169 |
| rs4648128 | Chr4:103536024 | 0.067 | G/A | 0.064 | 0.958 (0.718-1.279) | 0.775 | 0.046 | 0.674 (0.393-1.155) | 0.148 |
| rs230547 | Chr4:103536261 | 0.100 | T/C | 0.091 | 0.897 (0.702-1.147) | 0.386 | 0.103 | 1.031 (0.705-1.507) | 0.875 |

**Supplementary figure S1: Scatter plot showing the principal component analysis (PCA) in our Spanish samples compared with different populations in HapMap. The eigenvalues for the first three principal components accounted for most of the population substructure in this analysis (77.5%). All individuals who were not clustering with the main cluster (> 3 Standard deviation from cluster center) were excluded from subsequent analysis. Using this method we identified a total of 48 outliers individuals in our case-control cohort. X-axis represents Principal Component 1 (PC1) and Y-axis represents Principal Component 3 (PC3) in our Spanish samples (diamonds), and the main populations in HapMap: CEU, Northern European from Utah (squares), CHB+JPB, Chinese in Beijing+ Japanese in Tokyo (triangles), MEX (crosses), TSI, Tuscans from Italy (asterisks) and YRI, Yoruba in Ibadan, Nigeria (circles).**

**
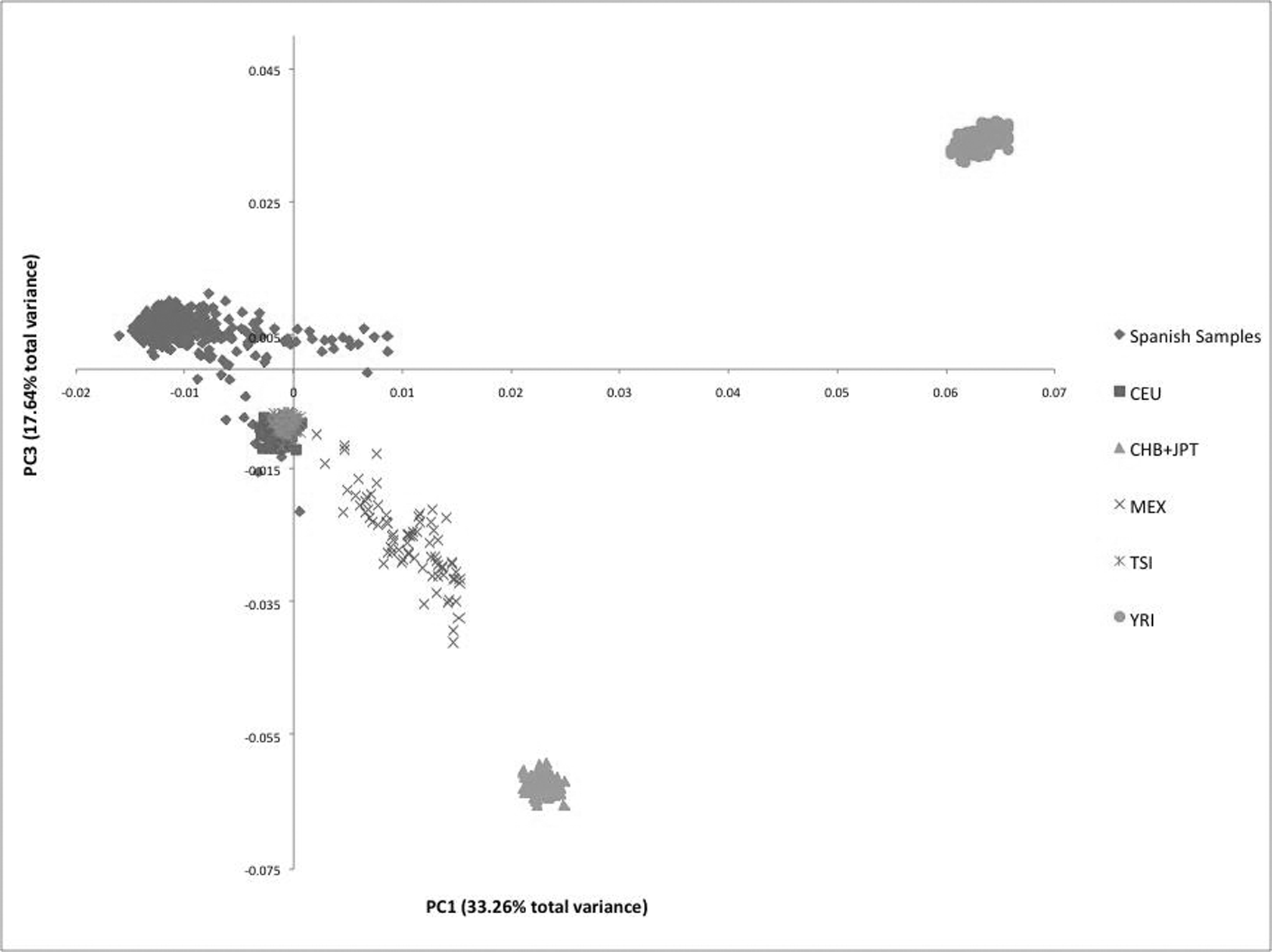
**
